# Detection of IgG Anti-*Leishmania* Antigen by Flow Cytometry as a Diagnostic Test for Cutaneous Leishmaniasis

**DOI:** 10.1371/journal.pone.0162793

**Published:** 2016-09-13

**Authors:** Geraldo Pedral-Sampaio, Jessé S. Alves, Albert Schriefer, Andréa Magalhães, Roberto Meyer, Marshall J. Glesby, Edgar M. Carvalho, Lucas P. Carvalho

**Affiliations:** 1 Laboratório de Imunologia, Instituto de Ciências da Saúde, Universidade Federal da Bahia, Salvador, Brazil; 2 Serviço de Imunologia, Complexo Hospitalar Professor Edgard Santos, Universidade Federal da Bahia, Salvador, Brazil; 3 Division of Infectious Diseases, Weill Cornell Medical College, New York, United States of America; 4 Instituto Nacional de Ciências e Tecnologia - Doenças Tropicais – INCT-DT, Salvador, Brazil; 5 Laboratório Avançado de Saúde Pública, Fiocruz – BA, Salvador, Brazil; Ohio State University, UNITED STATES

## Abstract

Diagnosis of cutaneous leishmaniasis (CL) relies on clinical presentation, parasite isolation, histopathologic evaluation and positive Montenegro skin test. However, the low amounts of parasites in the lesion of these individuals make parasite isolation and histopatologic diagnosis unreliable, often leading to false-negative results. Also, 15% of people living in endemic areas have sub-clinical infection characterized by positive Montenegro skin test, which may contribute to misdiagnosis. Although the main *Leishmania* killing mechanism is through cell-mediated immune response, antibodies against *Leishmania* antigens are found in infected individuals. Here our goal was to develop a new serological technique using polystyrene microspheres sensitized with soluble *Leishmania* antigens as a tool for the detection of IgG in serum from CL patients by flow cytometry. To validate the assay we carried out a comparative test (ELISA) commonly used as a diagnostic test for parasitic diseases. To determine cross-reactivity we used serum from patients with Chagas disease, caused by a trypanosome that has several proteins with high homology to those of the *Leishmania* genus. We observed that the flow cytometry technique was more sensitive than the ELISA, but, less specific. Our results show that the flow cytometry serologic test can be used to confirm CL cases in *L*. *braziliensis* transmission areas, however, presence of Chagas disease has to be ruled out in these individuals.

## Introduction

Cutaneous leishmaniasis (CL) caused by *Leishmania braziliensis* is characterized by the presence of one or more well-delineated ulcerated lesions that is mainly composed of lymphocytes, mononuclear phagocytes and plasma cells [1, 2]. In CL patients the immune response is predominantly mediated by mononuclear cells, which involve mechanisms associated with delayed type hypersensitivity with production of IFN-gamma and TNF [3–5]. This kind of response mediates parasite killing through activation of macrophages and also leads to tissue damage observed in these individuals [5].

The diagnosis of CL is mainly based on clinical observations and *Leishmania* skin test; histopathologic or PCR techniques are usually used as confirmatory tests [6–9]. However, due to the low frequency of parasites in lesions of *L*. *braziliensis*-infected individuals, the use of PCR may lead to false-negative results. The contribution of the humoral immune response to protection, immunopathology or prevention of parasite dissemination in leishmaniasis is controversial and not well established, although, presence of antibodies during active disease have encouraged the development of serological tests for diagnosis and epidemiological investigations [10–14]. Varying profiles and levels of antibodies against *Leishmania* have been detected in CL patients, mainly due to differences in parasitic load, species involved, time since infection and intrinsic host factors [15–18].

Methods to evaluate the humoral immune response are mainly based on *in vitro* serologic surveys using soluble antigens, recombinant antigens and fixed parasites, such as indirect immunofluorescence, indirect hemaglutination and ELISA. Problems with the analysis of antibody titers by conventional serologic methods to detect *Leishmania* infection include cross-reactivity with other species of the Trypanosomatidae family, low sensitivity and lack of association with the presence of active infection [19, 20].

Serological studies based on flow cytometry using polystyrene microspheres coated with soluble antigens constitute a field with growth potential due to the increased sensitivity of this method [21, 22]. In the present study we have developed a serological technique using polystyrene microspheres sensitized with soluble *Leishmania* antigen (SLA) for the detection of IgG antibodies in the serum of CL patients by flow cytometry and have compared this with an ELISA test. We show that the flow cytometry-based test has greater sensitivity compared to the ELISA test, though neither test has the capacity to distinguish between samples from *T*. *cruzi* and *L*. *braziliensis* infected individuals.

## Materials and Methods

### Patients

Participants of this study were from the Corte de Pedra endemic area in Northeastern Brazil, a *L*. *braziliensis* transmission area where more than 1000 cases are diagnosed per year. The study population consisted of 27 CL patients, 26 household contacts of CL patients, with evidence of exposure to *Leishmania* but without disease, 9 individuals with Chagas disease and 10 healthy subjects living in a non-endemic area. Leishmaniasis patients were diagnosed based on clinical presentation compatible with cutaneous leishmaniasis, positive Montenegro skin test and parasite isolation. Chagas disease patients were diagnosed by a serologic test to detect IgG to *Trypanosoma cruzi* (Diagnostic Automation, INC, CA, USA). Individuals with evidence of exposure to *Leishmania* but without disease were identified by positive delayed type hypersensitivity (DTH—Montenegro skin test), IFN-gamma production to SLA and absence of lesions or history of leishmaniasis. All blood samples were collected before treatment of CL or Chagas disease had been started. To determine sensitivity, specificity, positive and negative predictive value we used 2 by 2 contingency tables containing: true positive; false positive; true negative; false negative (Tables 1, 2 and 3). The number of true positive, false positive, true negative and false negative individuals from each group analysed are represented on Tables 2 and 3. This study was approved by ethical committee of the University Hospital at the Federal University of Bahia. Written informed consent was obtained from all participants.

**Table 1:** Representative table and formulas used to calculate diagnostic tests performance.

Test	Disease present	Disease absent
Positive	a- True positive	c- False positive
Negative	b- False negative	d- True negative

Sensitivity = a / (a+b). Specificity = d / (c+d). Positive predictive value = a / (a+c). Negative predictive value = d / (b+d)

**Table 2:** Number of individuals according to each parameter used for calculation of ELISA test performance.

	CL × HS	CL × DTH+	CL × CD
True positive	27	27	27
True negative	8	8	0
False positive	2	18	9
False negative	0	0	0

CL, cutaneous leishmaniasis; HS, healthy subjects; DTH+, delayed type hypersensitivity positive; CD, Chagas disease.

**Table 3:** Number of individuals according to each parameter used for calculation of Flow cytometry test performance.

	CL × HS	CL × DTH+	CL × CD
True positive	27	27	27
True negative	10	23	0
False positive	0	3	9
False negative	0	0	0

CL, cutaneous leishmaniasis; HS, healthy subjects; DTH+, delayed type hypersensitivity positive; CD, Chagas disease.

### Indirect ELISA

Specific anti-*Leishmania* IgG was measured by ELISA as follows: highly sensitive microplates (Thermo scientific, Waltham, USA) were sensitized with 100μl of 20μg/ml soluble antigen of *L*. *(V*.*) braziliensis* and incubated at 4°C overnight. The plates were then washed five times with PBS-Tween and incubated with 100 μl/well of each individual’s serum diluted 1:100 to 1:800 in 1x PBS for 1 hour at 37°C. After washing three times with PBS-Tween, 100 μl/well of anti-human IgG (γ-chain specific) was added and plates were incubated at 37°C for 1 hour and washed three times with PBS-Tween. Alkaline phosphatase conjugate antibody class detection monoclonal antibody (Sigma A-3150), diluted 1:500 in PBS-Tween 0.05%, was added and plates were incubated for 1 hour at 37°C. After the plates had been washed three times, enzymatic activity was developed by incubation with p-nitrophenyl phosphate (Sigma). Absorbance was read at 405 nm in a microplate reader (BioRad). To determine positivity of the samples, the cutoff was determined by a ROC curve containing absorbances from CL patients and healthy subjects.

### Microspheres treatment

Polystyrene microspheres (SPHERO^™^ Polystyrene Particles, Chicago, EUA) of 8μm diameter were sensitized with soluble *Leishmania* antigen (SLA) as follows: PBS1x (1.8 ml), 0.2 ml of SLA (1mg/ml), and 0.2 ml of microsphere were mixed together in a conical tube and incubated for 1 hour at room temperature, protected from light. Thereafter, the mixture underwent centrifugation at 3,000g for 15 minutes at 25°C and supernatant was removed carefully. The pellet was then resuspended in 4 ml of PBS1x solution and centrifuged as above. After centrifugation the supernatant was carefully removed and the pellet was re-suspended in 4 ml of PBS1×. As negative controls for the experiments we used microspheres that were not coated with SLA and performed the same protocol described above.

### Flow cytometry

**Fig 1:**
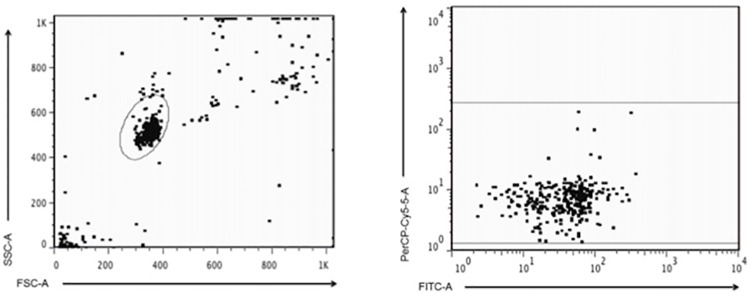
Gate strategy to determine beads population to be analyzed. A gate was performed based on FSC and SSC and than mean florescence intensity was assessed based on FITC staining.

For the flow cytometry assays we used 96-well "U" bottom containing 20μl of serum diluted in PBS (1:100 to 1:800) plus 20μl of the suspension of microspheres sensitized with the SLA as described above. The mixtures were incubated at 37°C for 20 min, washed with 300μl of 1x PBS by centrifugation (4,000 rpm, 5 min), and the supernatants discarded. To detect anti-SLA IgG on the microsphere surfaces we added to the mixture above 20μl of anti-IgG polyclonal antibody (specific for Fc portion) labeled with fluorescein isothiocyanate—FITC (Sigma-Aldrich, Co. St. Louis, MO, USA), diluted 1:500 in 1x PBS. Samples were then incubated at 37°C for 20 minutes, protected from the light. After the incubation period, samples were washed (300μl of 1x PBS at 4,000 rpm for 5 min) and supernatants were carefully discarded. Samples were acquired on FACS Canto II flow cytometer (Becton Dickinson, San Jose, USA) and analysis was performed using the software Flowjo (Tree Star, Inc. OR, USA). The analysis consisted of the selection of the gate containing the microspheres, based on size and complexity, and the mean fluorescence intensity (MFI) of the FITC channel (Fig 1). To determine positivity of the samples, the cutoff was determined by a ROC curve containing MFI from CL patients and healthy subjects.

## Results

**Fig 2:**
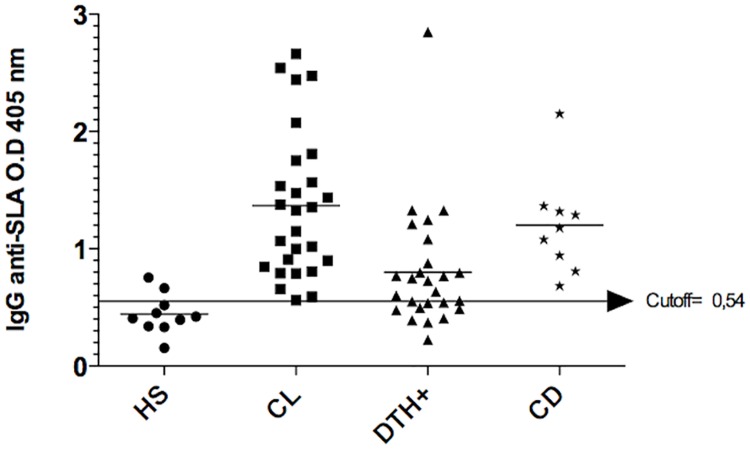
Antibody titers assessed by ELISA. Anti-SLA IgG titers from healthy subjects (HS), cutaneous leishmaniasis patients (CL), Montenegro positive individuals (DTH+) and Chagas disease patients (CD), assessed by ELISA. Cut off was determined by a ROC curve containing absorbances from CL patients and healthy subjects. OD, optical density.

**Fig 3:**
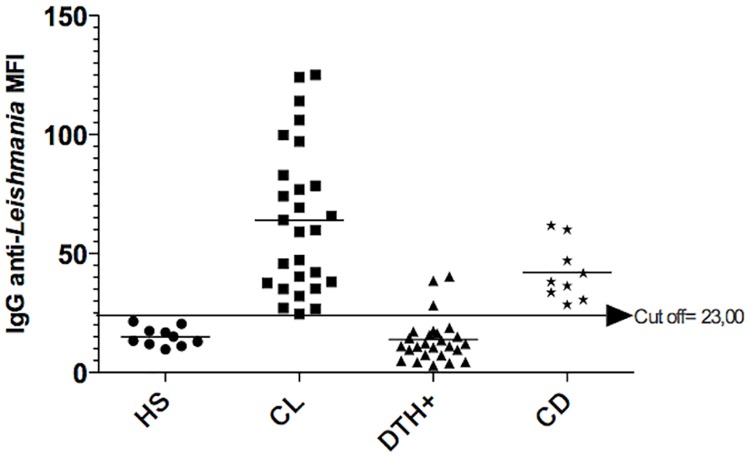
Antibody titers assessed by flow cytometry. Anti-SLA IgG titers from healthy subjects (HS), cutaneous leishmaniasis patients (CL), Montenegro positive individuals (DTH+) and Chagas disease patients (CD), assessed by flow cytometry. Cut off was determined by a ROC curve containing MFI from CL patients and healthy subjects. MFI, mean fluorescence intensity.

Forty-six serum samples as described above were used for standardization of the ELISA and flow cytometry tests. The cut–offs were 0.54 OD for ELISA and 23 MFI for flow cytometry. Based on the absorbance and MFI of the healthy subjects and CL patients, we found 100% sensitivity for both tests (Figs 2 and 3 and Table 4).

**Table 4:** Performance of ELISA and Flow cytometry tests to diagnose cutaneous leishmaniasis cases.

	ELISA	95% CI	Flow cytometry	95% CI
		(ELISA)		(Flow cytometry)
Sensitivity	100%	87.23% to 100%	100%	87.23% to 100%
Specificity CL X	0%	0% to 33.63%	0%	0% to 33.63%
CD				
Specificity CL X	30.77%	14.33% to 51.79%	88.46%	69.85% to 97.55%
DTH+				
PPV (DTH+)	60.0%	44.33% to 74.30%	90.0%	73.47% to 97.89%
NPV (DTH+)	100%	63.06% to 100%	100%	85.18% to 100%

CI, confidence interval; CL, cutaneous leishmaniasis; CD, Chagas disease; DTH+, delayed type hypersensitivity positive; PPV, positive predictive value; NPV, negative predictive value.

Chagas disease is endemic in Brazil and *T*. *cruzi* expresses proteins with high degree of homology to *Leishmania sp*., which may lead to false positive results in diagnostic tests for leishmaniasis based on antibody titers. Thus, we included serum from Chagas disease patients to determine the specificity of ELISA and flow cytometry tests. Both tests failed to distinguish between Chagas disease and CL, indicating that an ELISA for Chagas disease should always be performed if one of these tests is used for CL diagnosis (Figs 2 and 3 and Table 4).

Around 15% of healthy individuals living in *L*. *braziliensis* transmission areas have positive delayed type hypersensivity (DTH) to *Leishmania* antigens (Montenegro skin test) without clinical symptoms. Thus, we then investigated if our tests were able to discriminate between clinical active disease and healthy individuals with positive DTH. The performance of the flow cytometry test was better than the ELISA (specificity, 88.46% and 30.77%, respectively) (Figs 2 and 3 and Table 4). Moreover, the positive predictive value to distinguish between CL and DTH positive individuals was higher for the flow cytometry test (90%) when compared to the ELISA (60%), whereas, the negative predictive values were 100% for both tests (Table 4).

## Discussion

The diagnosis of CL is usually performed based on clinical features and confirmed by DTH test, accompanied or not by serological tests, histopathologic findings, *in vitro* growth of the parasite or molecular methods, such as PCR. A major problem of diagnostic methods that rely on the presence of the parasite is that the number of *Leishmania* organisms in CL lesions is very low. Thus, direct tests such as growth of the parasite in media and indirect ones, such as PCR, often lead to false negative results. Serological tests, however, usually have low sensitivity of antibody detection and high cross-reactivity with other diseases such as Chagas disease and leprosy [19, 23]. The ELISA has been widely used as a diagnostic tool for CL, but high variability of test performance characteristics has been observed. The variability is likely due to factors including differences in antigenic preparation, species of *Leishmania* and also clinical presentation of patients. Therefore, many investigators have attempted to increase the sensitivity and eliminate cross-reactivity of serological tests for the diagnosis of leishmaniasis [19, 20].

In a study comparing the performance of the ELISA with indirect immunofluorescence and immunoblotting using SLA from *L*. *amazonensis* and *L*. *braziliensis*, test performance was better when *L*. *braziliensis* antigen was used. The indirect immunofluorescence test showed low sensitivity (56.7%) for *L*. *amazonensis* antigen and high sensitivity (91.7%) for *L*. *braziliensis* antigen. The ELISA also showed low sensitivity (71.7%) for *L*. *amazonensis* SLA and a high sensitivity (95%) for *L*. *braziliensis* SLA. In contrast, the immunoblot test showed a high sensitivity for *L*. *amazonensis* and *L*. *braziliensis* SLA (83.3% and 96.6%, respectively) [24]. These data point to a better performance of the *L*. *braziliensis* SLA for the diagnosis of CL by different serological methods.

The flow cytometry technique has been used as a diagnostic and monitoring tool for infectious diseases and cancer [21]. In CL, flow cytometry assays have been used as diagnostic tests and to assess cure post therapeutic intervention [25–27]. Recently, a flow cytometry assay using SLA and live promastigotes was compared with the ELISA test for the diagnosis of patients with CL. In this study, although more sensitive, the flow cytometry test showed lower specificity when compared to the ELISA [28].

In the present study, we developed a technique using polystyrene beads coated with *L*. *braziliensis* SLA to detect anti-SLA IgG in human samples using flow cytometry and compared results of diagnostic testing of patients in an endemic area for *L*. *braziliensis* with an ELISA using the same antigen preparation. The results showed equivalent sensitivity for both tests (100%) and neither was able to distinguish between infection by CL and Chagas disease.

Cross-reactivity with other diseases, such as Chagas disease, toxoplasmosis and visceral leishmaniasis is one of the most frequent problems in studies of diagnostic tests for CL [20, 28–30]. One of the reasons for high rates of cross-reactivity in serological tests is due to the fact that many organisms share proteins with high degrees of homology. One strategy to decrease cross-reactivity is the use of subunits of organisms for diagnostic tests [23, 31]. In a study where an antigen fraction obtained from *L*. *braziliensis* promastigotes was used, investigators found a sensitivity of 85.41% and a specificity of 92.42% in comparison to other diseases, including Chagas disease, toxoplasmosis and paracoccidioidomycosis. However, this test had a high rate of false positive results among healthy controls (10.4%) [30]. Flow cytometry for the diagnosis of leishmaniasis has been compared with other tests in addition to the ELISA, such as an indirect immunofluorescence test. These studies also showed that the flow cytometry method had higher sensitivity and lower specificity in relation to indirect immunofluorescence [22, 32].

Most CL diagnostic studies do not address the ability of these tests to distinguish between active disease and exposure to *L*. *braziliensis* (based on DTH positivity). If we consider positivity of Montenegro skin test as a proxy of *L*. *braziliensis* infection the rate of infection to disease in *L*. *braziliensis* endemic areas is 3.7:1 [33]. As asymptomatic individuals are identified by positive DTH to *Leishmania* antigen, and CL patients also have positive DTH to *Leishmania* antigen, the chance of false positive tests is high. Therefore, another objective of our study was to determine whether our test was able to differentiate individuals with disease from those who are healthy with positive DTH. Here we found that the flow cytometry test was better able to discern between active infection and positive DTH alone than the ELISA test (specificity of 88.46% and 30.77%, respectively).

CL is an endemic disease in more than 80 countries and to date there is no gold standard technique for the diagnosis of this disease. Our study opens prospects for a new method using flow cytometry with high sensitivity albeit low specificity. One way to increase the specificity of this test may be the use of antigens that are specific to *Leishmania* pathogens and are recognized by antibodies of symptomatic patients. Future studies should test the flow cytometry serological test described herein using recombinant *Leishmania* antigens.
